# Prognostic risk profiling in COPD using Global Initiative for Chronic Obstructive Lung Disease 2023 ABE and comorbidity assessment: evidence from a register-based COPD cohort

**DOI:** 10.7189/jogh.15.04152

**Published:** 2025-05-23

**Authors:** Ching-Hsiung Lin, Yi-Rong Li, Shih-Lung Cheng, Hao-Chien Wang, Hen-I Lin, Kang-Yun Lee, Inn-Wen Chong, Po-Chiang Chan, Huan-Wei Chen, Chong-Jen Yu

**Affiliations:** 1 Division of Chest Medicine, Department of Internal Medicine, Changhua Christian Hospital, Changhua, Taiwan; 2 Institute of Genomics and Bioinformatics, National Chung Hsing University, Taichung, Taiwan; 3 PhD Program in Translational Medicine, National Chung Hsing University, Taichung, Taiwan; 4 Department of Post-Baccalaureate Medicine, College of Medicine, National Chung Hsing University, Taichung, Taiwan; 5 Thoracic Medicine Research Centre, Changhua Christian Hospital, Changhua, Taiwan; 6 Department of Internal Medicine, Far Eastern Memorial Hospital, Taipei, Taiwan; 7 Department of Chemical Engineering and Materials Science, Yuan Ze University, Taoyuan, Taiwan; 8 Department of Internal Medicine, National Taiwan University Hospital, Taipei, Taiwan; 9 Department of Internal Medicine, Cardinal Tien Hospital, New Taipei City, Taiwan; 10 Division of Pulmonary Medicine, Department of Internal Medicine, Shuang Ho Hospital, Taipei Medical University, New Taipei City, Taiwan; 11 Division of Pulmonary and Critical Care Medicine, Department of Internal Medicine, Kaohsiung Medical University Hospital, Kaohsiung, Taiwan; 12 Department of Biological Science and Technology, National Yang Ming Chiao Tung University, Hsinchu, Taiwan; 13 Department of Internal Medicine, National Taiwan University Hospital, Hsin-Chu Branch, Hsinchu County, Taiwan

## Abstract

**Background:**

While the Global Initiative for Chronic Obstructive Lung Disease (GOLD) 2023 ABE classification system guides initial chronic obstructive pulmonary disease (COPD) treatment, patient heterogeneity and comorbidities complicate management. We investigated how the GOLD 2023 ABE classification and aligned comorbidity profiles affect patient outcomes in real-world Asian populations with COPD.

**Methods:**

We conducted a register-based cohort study of 38 928 patients from multiple institutions across Taiwan (from April 2017 to December 2021). We classified patients by GOLD 2023 ABE categories. Data included demographics, Charlson comorbidity index (CCI)-defined comorbidities, treatment, symptoms, questionnaires, spirometry, and outcomes.

**Results:**

Among COPD patients, 89.2% were males, and the median age was 71 years. Groups A comprised 30.2%, group B 46.4%, and group E 23.5% of patients. Among these, 28.3% of group A patients used inhaled corticosteroid-containing inhalers. Group E had the highest rates of GOLD 4 airway obstruction (11.8%), CCI score ≥4 (15.6%), and five-year mortality rate (22.6%). Group E demonstrated the highest risk of all-cause mortality (hazard ratio (HR) = 1.727; 95% confidence interval (CI) = 1.605–1.858) and moderate-to-severe exacerbation (HR = 2.127; 95% CI = 1.942–2.330) *vs.* group A. Key comorbidities, acute myocardial infarction (HR = 1.257; 95% CI = 1.057–1.430), congestive heart failure (HR = 1.836; 95% CI = 1.707–1.909), and pulmonary disease (HR = 1.071; 95% CI = 1.011–1.129), were associated with higher mortality. Acute myocardial infarction (HR = 1.251; 95% CI = 1.031–1.444), congestive heart failure (HR = 1.193; 95% CI = 1.089–1.285), and pulmonary disease (HR = 1.491; 95% CI = 1.405–1.550) were also associated with higher exacerbations, with patterns varying across GOLD groups.

**Conclusions:**

In this large registry-based cohort, group E patients demonstrated the highest mortality and exacerbation risks. Cardiovascular and pulmonary comorbidities increased adverse outcome risks across all GOLD categories. Systematic comorbidity screening should be integrated into routine COPD care. Findings support personalised treatment approaches based on GOLD classification and comorbidity profiles.

Chronic obstructive pulmonary disease (COPD) is a heterogeneous and multisystemic lung condition that poses a significant global health burden as the third leading cause of death worldwide [1–3]. It is characterised by persistent airflow limitation and chronic respiratory symptoms caused by abnormalities in the airways and/or alveoli, leading to progressive airflow obstruction [4,5]. COPD significantly impairs patients’ quality of life and is frequently accompanied by multimorbidity, including other chronic conditions linked through shared risk factors such as smoking, ageing, and environmental exposures [6,7]. The characteristics and prognosis of COPD patients are continually evolving due to demographic shifts, such as an ageing population, and advancements in treatment strategies. Thus, understanding the most current baseline data and prognosis of COPD provides crucial insights into disease behaviour and supports precision management strategies.

The Global Initiative for Chronic Obstructive Lung Disease (GOLD) strategy provides guidelines for the prevention, diagnosis, treatment, and follow-up of COPD patients [8]. The COPD classification system has evolved significantly through several iterations. GOLD 2011 introduced a multidimensional ABCD system combining spirometry, symptoms, and exacerbation risk, but faced criticism for its complexity and potential for misclassification [9,10]. GOLD 2017 simplified assessment by removing spirometric thresholds from the ABCD grouping, focusing instead on symptoms and exacerbation history. Although previous studies have demonstrated that the GOLD ABCD grade effectively predicts future exacerbations and mortality in COPD patients, growing evidence indicates that exacerbation history remains the strongest predictor of future exacerbation risk and death [11,12]. The 2023 update further refined the classification system by introducing three subgroups A, B, and E, with a significant change merging the high-risk groups C and D into a single category E, defined solely by exacerbation history [5,13]. This change simplifies clinical assessment and optimises treatment recommendations. While these classifications were originally designed to guide treatment, they have been widely adopted for prognostic predictions [14–16], with previous studies revealing higher mortality and exacerbation rates in groups B and D [15,16]. However, despite these modifications, research in a recent study revealed that the GOLD 2023 classification based on ABE groups did not predict mortality better than the earlier 2017 ABCD classifications [17].

Comorbidities significantly influence COPD prognosis and are highly prevalent among COPD patients. These concurrent conditions – including cardiovascular diseases, metabolic disorders, mental health issues, musculoskeletal disorders, and cancer – share common risk factors and systemic mechanisms with COPD [6,7]. Their presence increases the frequency of exacerbations, hospitalisations, and mortality risk [18–20], as measured by validated indices such as the Charlson comorbidity index (CCI), body-mass index, obstruction, dyspnea, and exercise (BODE), and COPD-specific comorbidity test indices [19,21]. The complex interplay between COPD and comorbidities contributes to increased health care utilisation and reduced quality of life [22,23]. Effective management of COPD, therefore, requires an integrated approach that addresses both the primary respiratory condition and its associated comorbidities to improve patient outcomes. As global populations age, particularly in East Asian countries like Taiwan [24,25], COPD patients increasingly present with multiple comorbidities, including cardiovascular diseases, diabetes, and depression. This evolving patient profile necessitates comprehensive disease management strategies that extend beyond respiratory care, highlighting the importance of integrated approaches. However, large-scale real-world data on the characteristics, comorbidities, and prognosis of COPD under the GOLD 2023 ABE classification remain limited, particularly in ageing populations with increasing comorbidity burdens.

Real-world data offers a cost-effective, comprehensive source of patient insights, improving research generalisability and enabling the identification of long-term trends. While these data provide valuable opportunities to generate evidence on drug treatment effects in populations typically excluded from clinical trials, there remains a critical gap in validated, rigorous methodological approaches for conducting such analyses effectively [26–28]. Patient registries serve as a vital source of real-world data and real-world evidence. They can be precisely designed according to specific requirements, serving as a significant medium for observing actual clinical practices. With established quality standards and evaluation criteria, patient registries provide a solid foundation for research and development in health care, making them the new gold standard for real-world study [29–31]. Despite the growing use of real-world data for COPD research, including registry data, over the past decades, large-scale longitudinal registry data sets that align their data elements with existing COPD-specific guidelines remain scarce.

Taiwan COPD patient registry, part of the ongoing COPD integrated care initiative known as the Taiwan COPD Pay-for-Performance Program, is a five-year longitudinal data set designed in alignment with COPD-specific guidelines. In this study, we aimed to use data from the Taiwan COPD patient registry to investigate the baseline characteristics, comorbidity profiles, and long-term prognosis of spirometry-confirmed COPD patients enrolled in a guideline-based care program, classified according to GOLD 2023 classification.

## METHODS

### Study design and data source

We conducted this retrospective study using a Taiwan COPD national registry database, which is part of an ongoing longitudinal project known as Taiwan COPD pay-for-performance (P4P) and launched by the Taiwan National Health Insurance on 1 April 2017, aiming to provide an integrated disease management program for COPD patients. We used the Taiwan National Health Insurance claim database to gather demographic information, diagnostic codes, and health insurance claims data for patients enrolled in the Taiwan COPD P4P program. Between 1 April 2017 and 31 December 2021. This cohort consisted of approximately 40 000 participants from 216 primary and secondary care practices distributed throughout Taiwan, including medical centres, regional hospitals, district hospitals, and primary care clinics across diverse geographic regions. This multi-level health care facility recruitment approach ensures broad population representation beyond single-institution limitations. Moreover, the program encompasses multiple medical specialities, including family medicine, pulmonology and critical care, internal medicine, paediatrics, otolaryngology, and neurology, ensuring diverse clinical perspectives in patient management. All participating health care providers received standardised training on the Taiwan COPD P4P program and followed established Taiwan’s COPD guidelines with a disease management protocol.

We conducted data collection through a unified, nationwide virtual private network system with structured fields for case information registration, enhancing data consistency and minimising documentation variability. The P4P program also incorporates a systematic referral network allowing for upward referral of exacerbated cases and downward referral of stabilised patients, facilitating appropriate care levels regardless of initial recruitment site. The full details of the protocol of Taiwan’s COPD P4P program have been previously published. We adhered to the principles outlined in the Declaration of Helsinki, and the Institutional Review Board of Changhua Christian Hospital approved the study (approval number 230512). Informed consent was waived due to the retrospective design of the study. We conducted all procedures in compliance with relevant ethical guidelines and regulations. The data used in the study were de-identified, and researchers strictly followed Taiwan’s computer-processed personal data protection law and related privacy regulations.

### Participants and measurement method

Patients diagnosed with COPD (International Classification of Diseases (ICD-10) codes J41–J44) within the past 90 days at the same health care institution and confirmed by spirometry (forced expiratory volume in one second (FEV_1_)/forced vital capacity (FVC)<0.7) were eligible for inclusion. To qualify, we required patients to have at least two visits to the institution, with COPD documented as the primary diagnosis during the recruitment visit. We excluded patients with missing baseline data, including demographic information, spirometry results, the COPD assessment test (CAT) scores, and modified British Medical Research Council (mMRC) grades. All patients were classified according to GOLD 2023 guidelines.

### Spirometric measurements

We performed all spirometry according to the American Thoracic Society and European Respiratory Society standardisation guidelines. We collected FEV_1_, FVC, and their predicted values with appropriate adjustments for age, gender, body height, weight, and race [32]. These standardised procedures guarantee the reproducibility and accuracy of our spirometric data.

### Patient-reported outcomes

We measured patient-reported outcomes by the mMRC dyspnoea scale and the CAT. mMRC is a well-established five-point scale (zero to four) that assesses breathlessness during physical activities [33]. CAT is a comprehensive eight-question instrument (on a one to five point scale) that evaluates the overall impact of COPD on patients, including symptoms such as cough, sputum, breathlessness, chest tightness, and effects on confidence, activity, sleep, and energy levels [34]. Both the mMRC and CAT are validated questionnaires recommended by the GOLD guidelines for classifying COPD risk groups. Their reliability and validity have been established through extensive previous research [35,36]. In our previous study, CAT and mMRC have been found to provide individual benefits in the evaluation of clinical symptoms, comorbidities, and medical resource utilisation for emergency department visits, hospitalisation, and intensive care unit admission in Taiwanese COPD patients [37]. Given their proven reliability and clinical utility, we selected these validated measures to appropriately assess patient-reported outcomes in our research.

### Data collection and outcomes

Demographic data included age, gender, body mass index (BMI), and smoking status. Baseline clinical characteristics encompassed post-bronchodilator spirometry results, CCI score, severity of airflow limitation, mMRC grade, CAT score, acute exacerbation history, GOLD risk group classification, wheezing, family history of COPD, and maintenance inhaler therapy. We determined airflow limitation severity and GOLD risk group classifications according to the 2023 GOLD guidelines. We categorised airflow limitation as follows: mild (FEV_1_≥80% predicted), moderate (50%≤FEV_1_<80% predicted), severe (30%≤FEV_1_<50% predicted), and very severe (FEV_1_<30% predicted). We used the CCI to assess baseline comorbidity burden. We defined comorbidities as present if the relevant code appeared at least once in admission records or three times in outpatient department diagnosis codes [38]. Moreover, we applied the well-validated ICD coding algorithm based on the claims-based, disease-specific refinements, matching translation to ICD-10, flexibility (CDMF) to allow use as a chart review instrument CCI scheme described by Glasheen et al [39]. We excluded from the analysis cases with incomplete demographic data, baseline spirometry measurements, or CAT/mMRC scores. As clinical covariates were fully accessible through the electronic medical record system for all admitted patients, we employed a complete case analysis approach, ensuring that we included in the final analysis only patients with complete data across all variables. The primary outcome was all-cause mortality, while the secondary outcome was moderate-to-severe exacerbations. Moderate exacerbations were defined as prescriptions of short-acting bronchodilators and oral corticosteroids (anatomical therapeutic chemical codes H02AB and R03CC) combined with a diagnosis code for COPD (ICD-10 codes J41–J44) on the same day, ensuring the medication was prescribed specifically for COPD exacerbation [40]. Severe exacerbations were identified through emergency department visits and hospital admissions lasting more than two consecutive days with a primary diagnosis of COPD [41]. We obtained mortality data from National Health Insurance disenrollment records marked as death with recorded dates.

### Statistical analysis

We reported descriptive data as medians (MD) and interquartile ranges (IQR) or percentages, as appropriate. We performed comparisons between groups for descriptive summaries using χ^2^ tests for categorical variables and analysis of variance for continuous variables. Given the concern over the three, rather than two groups in our study, we used inverse probability weighting (propensity score) for each patient via logistic regression to reduce the confounding effects due to the imbalances in the distribution of baseline characteristics. A multivariable Cox proportional hazards model was used to establish independent predictors of all-cause mortality and moderate-to-severe exacerbation. For moderate-to-severe exacerbations, we employed competing risk analysis to account for death as a competing event. We presented results as adjusted hazard ratios (HRs) with corresponding confidence intervals (CIs). For data management, analysis, and visualisation, we used SAS, version 9.4 (SAS Institute Inc., Cary, North Carolina, USA) or *R*, version 4.1.0 (R Core Team, Vienna, Austria). We considered a two-tailed *P*-value <0.05 statistically significant in all analyses.

## RESULTS

### Baseline characteristics and clinical outcomes of COPD patients by GOLD 2023 classification

We analysed 38 928 COPD patients categorised by GOLD 2023, following their reclassification from the previous GOLD 2017 system. The analysis showed that patients formerly in GOLD 2017 group C (n = 2977, 7.6%) and group D (n = 6154, 15.8%) were reclassified into the new GOLD 2023 group E (n = 9131, 23.5%) (Figure S1 in the **Online Supplementary Document**). Age was MD = 71 years, with group A being younger, and groups B and E older. Most patients were predominantly male. Smoking history varied significantly, with group E having the highest proportion of former smokers (59.4%). Pulmonary functions, including FEV_1_, the percentage predicted forced expiratory volume in one second (FEV_1%_ predicted) and FEV_1_/FVC ratios, were highest in group A and lowest in group E. Airflow limitation was primarily moderate, with group E having the highest proportion of severe obstruction (stage four). Group E also showed a higher symptom burden (mMRC and CAT scores), proportion of wheezing and more severe comorbidities compared to other groups.

The proportion of patients without a prescription decreased across all groups, with group A having the highest rate. Among all groups, the use of long-acting muscarinic antagonists (LAMAs) was higher compared to long-acting beta-agonists (LABAs). LABA/LAMA combination was the most frequently prescribed in the overall cohort (43.4%) as well as each group (39.0% in group A, 47.4% in group B, and 39.6% in group E), whereas the triple inhaler was most commonly used in group E (34.3%). The proportion of patients using dual inhalers increased progressively across groups A (66.5%), B (76.3%), and E (86.8%). Furthermore, 28.3% of group A patients using inhaled corticosteroid-containing inhalers. The mortality rate over five years was 14.3% of overall patients, highest in group E (22.6%) and lowest in group A (8.4%) (**Table 1**).

**Table 1:** Baseline demographic and outcome of patients with COPD by GOLD 2023 classification*

Demographics	All patients (n = 38 928)	Group A (n = 11 741)	Group B (n = 18 056)	Group E (n = 9131)	*P*-value
Age in years, MD (IQR)	71 (64–78)	69 (63–77)	72 (64–79)	72 (64–79)	<0.0001
Age category in years					<0.0001
*≤50*	987 (2.5)	371 (3.2)	411 (2.3)	205 (2.2)	
*51–60*	4639 (11.9)	1609 (13.7)	2016 (11.2)	1014 (11.1)	
*61–70*	12 104 (31.1)	4057 (34.6)	5317 (29.4)	2730 (29.9)	
*71–80*	12 771 (32.8)	3768 (32.1)	6069 (33.6)	2934 (32.1)	
*>80*	8427 (21.6)	1936 (16.5)	4243 (23.5)	2248 (24.6)	
Male	34 722 (89.2)	10 506 (89.5)	16 074 (89)	8142 (89.2)	<0.0001
BMI in kg/m^2^, MD (IQR)	23.7 (21.2–26.4)	23.9 (21.5–26.4)	23.8 (21.2–26.5)	23.3 (20.6–26.2)	<0.0001
BMI category in kg/m^2^					<0.0001
*<18.5*	3005 (7.7)	698 (5.9)	1329 (7.4)	978 (10.7)	
*18.5–24*	17 470 (44.9)	5255 (44.8)	8026 (44.5)	4189 (45.9)	
*≥24*	18 453 (47.4)	5788 (49.3)	8701 (48.2)	3964 (43.4)	
Smoking status					<0.0001
*Never*	6202 (15.9)	2016 (17.2)	2851 (15.8)	1335 (14.6)	
*Former*	21 255 (54.6)	6124 (52.2)	9704 (53.7)	5427 (59.4)	
*Current*	11 471 (29.5)	3601 (30.7)	5501 (30.5)	2369 (25.9)	
COPD family history					<0.0001
*Yes*	3120 (8)	832 (7.1)	1394 (7.7)	894 (9.8)	
*No*	35 808 (92)	10 909 (92.9)	16 662 (92.3)	8237 (90.2)	
Pulmonary function test, MD (IQR)					
*Post-BD FEV_1_, L*	60.0 (50.4–67.0)	66.0 (50.0–81.0)	59.0 (45.0–74.0)	47.0 (35.0–63.0)	<0.0001
*Post-BD FEV_1_, % predicted*	1.4 (1.03–1.85)	1.56 (1.15–2.01)	1.32 (0.97–1.74)	1.06 (0.78–1.43)	<0.0001
*Post-BD FEV_1_/FVC, %*	62.0 (46.7–78.0)	62.8 (54.4–68.3)	59.8 (50.4–66.2)	55 (45.2–63.8)	<0.0001
mMRC dyspnea scale, MD (IQR)	2 (1–2)	1 (0–1)	2 (1–2)	2 (1–3)	<0.0001
mMRC category					<0.0001
*<2*	18 744 (48.2)	10 866 (92.5)	5367 (29.7)	2511 (27.5)	
*≥2*	20 184 (51.8)	875 (7.5)	12 689 (70.3)	6620 (72.5)	
CAT score, MD (IQR)	10 (6–16)	5 (3–8)	12 (10–17)	13 (9–20)	<0.0001
CAT category					<0.0001
*1–10*	20 189 (51.9)	10 762 (91.7)	6207 (34.4)	3220 (35.3)	
*11–20*	14 650 (37.6)	754 (6.4)	9854 (54.6)	4042 (44.3)	
*21–30*	3742 (9.6)	194 (1.7)	1888 (10.5)	1660 (18.2)	
*31–40*	347 (0.9)	31 (0.3)	107 (0.6)	209 (2.3)	
Wheezing					<0.0001
*Yes*	7378 (19)	1043 (8.9)	3593 (19.9)	2742 (30)	
*No*	31 550 (81)	10 698 (91.1)	14 463 (80.1)	6389 (70)	
CCI, MD (IQR)	1 (0–2)	1 (0–2)	1 (0–2)	1 (0–3)	<0.0001
CCI category					
*0*	12 149 (31.2)	4057 (34.6)	5593 (31)	2499 (27.4)	
*1–3*	21 265 (54.6)	6230 (53.1)	9830 (54.4)	5205 (57)	
*≥4*	5514 (14.2)	1454 (12.4)	2633 (14.6)	1427 (15.6)	
Exacerbation history in the prior year					<0.0001
*<2*	34 150 (87.7)	11 566 (98.5)	17 470 (96.8)	5114 (56)	
*≥2*	4778 (12.3)	175 (1.5)	586 (3.2)	4017 (44)	
Airflow limitation severity level					<0.0001
*I*	8846 (22.7)	3776 (32.2)	3977 (22)	1093 (12)	
*II*	18 520 (47.6)	5681 (48.4)	9291 (51.5)	3548 (38.9)	
*III*	9254 (23.8)	1918 (16.3)	3921 (21.7)	3415 (37.4)	
*IV*	2308 (5.9)	366 (3.1)	867 (4.8)	1075 (11.8)	
Maintenance inhaler treatment					<0.0001
*None*	1564 (4.0)	753 (6.4)	556 (3.1)	255 (2.8)	
*ICS*	192 (0.5)	89 (0.8)	52 (0.3)	51 (0.6)	
*LABA*	807 (2.1)	333 (2.8)	407 (2.3)	67 (0.7)	
*LAMA*	6861 (17.6)	2762 (23.5)	3269 (18.1)	830 (9.1)	
*LABA + ICS or LABA/ICS*	5107 (13.1)	1617 (13.8)	2312 (12.8)	1178 (12.9)	
*LABA + LAMA or LABA/LAMA*	16 752 (43.0)	4574 (39)	8559 (47.4)	3619 (39.6)	
*Triple†*	7645 (19.6)	1613 (13.7)	2901 (16.1)	3131 (34.3)	
Five-year cumulative all-cause mortality rate					<0.0001
*Yes*	5578 (14.3)	988 (8.4)	2528 (14)	2062 (22.6)	
*No*	33 350 (85.7)	10 753 (91.6)	15 528 (86)	7069 (77.4)	

BMI – body mass index, CAT – COPD assessment test, CCI – Charlson comorbidity index, COPD – chronic obstruction pulmonary disease, FEV1 – forced expiratory volume in the first-second, FEV1%pred – percentage predicted the forced expiratory volume in one second, FVC – forced vital capacity, ICS – inhaled corticosteroid, IQR – interquartile range, LABA – long-acting β2-agonist, LAMA – long-acting muscarinic antagonist, MD – median, mMRC – modified Medical Research Council

*Presented as n (%) unless specified otherwise.

†Presented as LABA+LAMA+ICS or LABA/LAMA+ICS or LABA/LAMA/ICS.

### Survival analysis and risk factors for all-cause mortality and moderate-to-severe exacerbation across GOLD 2023 classification

**Figure 1:**
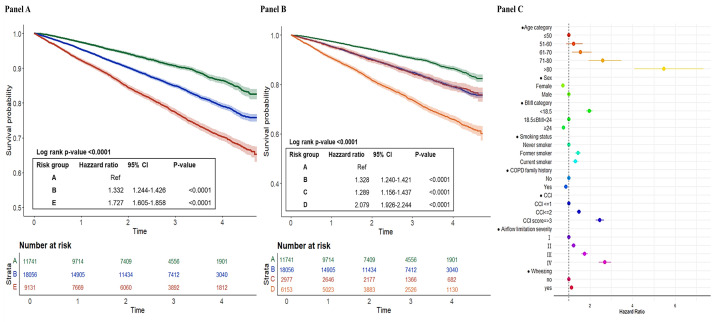
Kaplan-Meier curves and forest plot of all-cause mortality. **Panel A.** All-cause mortality by GOLD 2023 classification. **Panel B.** All-cause mortality by GOLD 2017 classification. **Panel C.** Risk factor of all-cause mortality.

The results demonstrate significant differences in outcomes across the risk groups (A, B, and E), with a notable severity gradient. Patients in group E experienced the lowest survival rate and the highest exacerbation rate when compared to Group A (*P* < 0.0001), suggesting a more pronounced disease burden in these patients. The HRs for five-year all-cause mortality were 1.3 times higher in group B (HR = 1.332; 95% CI = 1.244–1.426, *P* < 0.0001) and 1.7 times higher in group E (HR = 1.727; 95% CI = 1.605–1.858, *P* < 0.0001), relative to group A. This clearly highlights the increasing risk of death with worsening classification (**Figure 1**, Panel A).

**Figure 2:**
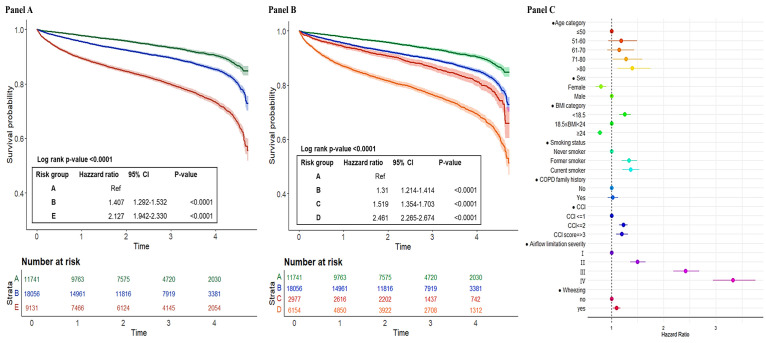
Kaplan-Meier curves and forest plot of moderate-to-severe exacerbation. **Panel A.** All-cause mortality by GOLD 2023 classification. **Panel B.** All-cause mortality by GOLD 2017 classification. **Panel C.** Risk factor of moderate-to-severe exacerbation

Interestingly, by applying the classification of GOLD 2017, the outcomes for patients in group B were found to be quite similar to those in group C, with their Kaplan-Meier curves nearly overlapping. Moreover, there was no significant difference in HRs for both all-cause mortality between groups B and C under GOLD 2017. On the other hand, a significant distinction was noted between groups C and D under GOLD 2017, with group D having significantly worse outcomes in all-cause mortality. The HRs for group D were notably higher, indicating that patients in this group faced a markedly greater disease burden (**Figure 1**, Panel B). The multivariable Cox proportional hazards regression results analysis indicated that the risk factors for five-year all-cause mortality included age >80 years, gender, smoking, wheezing, a higher CCI score, lower BMI, and worse airflow obstruction. A family history of COPD had a significantly negative association with five-year all-cause mortality (**Figure 1**, Panel C). For moderate-to-severe exacerbation, the HRs for moderate-to-severe exacerbations were found to be 1.4 times higher in group B (HR = 1.407; 95% CI = 1.292–1.532, *P* < 0.0001) and 2.0 times higher in group E (HR = 2.127; 95% CI = 1.942–2.330, *P* < 0.0001) compared to group A (**Figure 2**, Panel A). Age >80 years old, gender, smoking, family history of COPD, wheezing, a higher CCI score, lower BMI, and GOLD 4 airflow obstruction were significant risk factors for moderate-to-severe acute exacerbation. The patterns were similar across GOLD 2023 group ABE categories. (**Figure 2**, Panels B–C; Figure S2 in the **Online Supplementary Document**).

### Distribution of comorbidities in COPD patients

**Figure 3:**
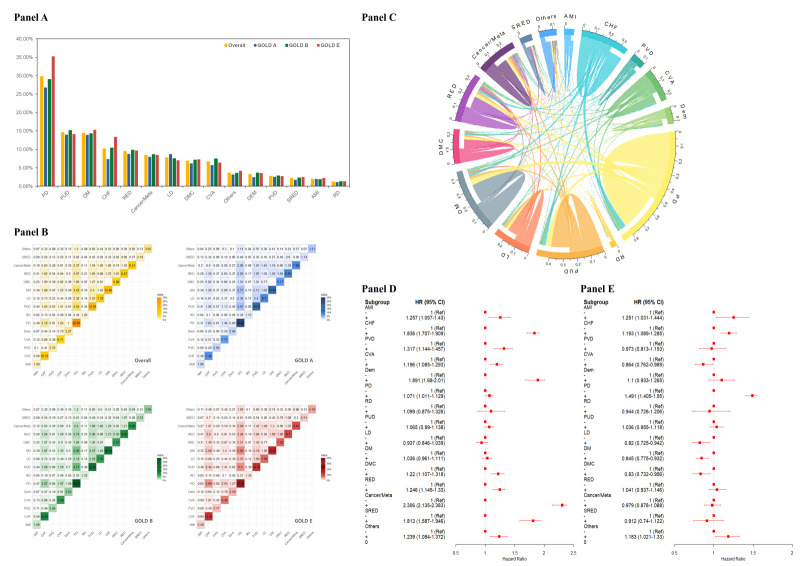
Distribution of the percentage of patients for each of the CCI diseases and the relationship between comorbidities, all-cause mortality, or moderate-to-severe exacerbations. **Panel A.** Distribution of the percentage of patients. **Panel B.** Percentage of patients diagnosed with two CCI diseases. The higher the percentage, the darker the colour of the heatmap cells. Gold, blue, green, and red cell colours correspond to all patients, GOLD A, GOLD B, GOLD E, respectively. **Panel C.** Chord diagram of the correlation between diseases. The length of each arc of the circle perimeter is proportional to the number of patients with the corresponding disease. **Panel D.** Forest plot of the relationship between comorbidities and all-cause mortality in patients with COPD. **Panel E.** Forest plot of the relationship between comorbidities and moderate-to-severe exacerbation in patients with COPD.

The analysis demonstrated significant variability in comorbidity prevalence between groups. Group E exhibited the highest percentage of patients with each comorbidity, while group A had the lowest burden of comorbid diseases. Among all groups, the three most prevalent CCI-defined comorbidities of COPD were pulmonary disease (PD), peptic ulcer disease (PUD), diabetes mellitus (DM) (**Figure 3**, Panel A). To gain a deeper understanding of the co-occurrence patterns of CCI-defined disease in COPD, we performed a comprehensive analysis on the frequency of all potential comorbidity pairs (**Figure 3**, Panel B). The findings revealed that most disease pairs have a co-occurrence frequency below 1.0%. Six diseases exhibited a co-occurrence rate exceeding 1.0% with other conditions, including congestive heart failure (CHF), PD, PUD, DM, renal disease (RED), and cancer/metastasis. The most prevalent co-occurrence was between PD and PUD (4.5%), followed by PD and DM (4.4%), as well as PD and CHF (3.1%). These patterns were consistently observed across all GOLD classification groups, reinforcing the robustness of these findings. Additionally, the chord diagram visualises a complex network of interactions among CCI-defined comorbidity (**Figure 3**, Panel C).

### Association between individual comorbidities and all-cause mortality among COPD

The forest plots illustrate specific mortality risks associated with comorbidities in COPD patients. The overall analysis (**Figure 3**, Panel D) revealed HRs for several key conditions: cancer/metastasis (HR = 2.306; 95% CI = 2.135–2.383), CHF (HR = 1.836; 95% CI = 1.707–1.909), dementia (HR = 1.891; 95% CI = 1.68–2.01), acute myocardial infarction (AMI) (HR = 1.257; 95% CI = 1.057–1.43), and PD (HR = 1.071; 95% CI = 1.011–1.129). In GOLD A patients (Figure S4, Panel A in the **Online Supplementary Document**), the HRs were: cancer/metastasis (HR = 2.344; 95% CI = 2.003–2.744), CHF (HR = 1.916; 95% CI = 1.622–2.267), diabetes with chronic complications (HR = 1.565; 95% CI = 1.286–1.905), and dementia (HR = 1.497; 95% CI = 1.118–2.005). For GOLD B patients (Figure S4, Panel B in the **Online Supplementary Document**), cancer/metastasis showed HR = 2.372 (95% CI = 2.116–2.659), CHF had HR = 1.736 (95% CI = 1.553–1.940), and peripheral vascular disease (PVD) demonstrated HR = 1.467 (95% CI = 1.206–1.785). The GOLD E cohort (Figure S4, Panel C in the **Online Supplementary Document**) exhibited the following HRs: cancer/metastasis (HR = 2.216; 95% CI = 1.909–2.551), CHF (HR = 1.92; 95% CI = 1.703–2.165), PVD (HR = 1.566; 95% CI = 1.237–1.981), dementia (HR = 1.858; 95% CI = 1.6–2.3), and AMI (HR = 1.121; 95% CI = 0.811–1.55). Common conditions such as DM maintained consistent HRs near 1.0 across all GOLD 2023 classifications. These findings demonstrate that cancer/metastasis and cardiovascular comorbidities, particularly CHF, are significant predictors of mortality in COPD patients across all GOLD 2023 classifications. Additionally, neurological conditions such as dementia show a substantial impact on mortality risk. This consistent pattern of risk across different GOLD classifications suggests that these comorbidities contribute to mortality independently of COPD 2023 classification.

### Association between individual comorbidities and moderate-to-severe exacerbation among COPD

We examined the relationship between comorbidities and moderate-to-severe exacerbation risk in COPD patients. In the overall analysis, PD showed significant association with exacerbation risk (HR = 1.491; 95% CI = 1.405–1.551), and AMI demonstrated an elevated risk (HR = 1.251; 95% CI = 1.031–1.444) (**Figure 3**, Panel E). Stratification by GOLD 2023 classification revealed specific patterns. Among GOLD A patients, PD remained a significant risk factor (HR = 1.387; 95% CI = 1.212–1.587), and CHF exhibited substantial risk (HR = 1.292; 95% CI = 1.041–1.602) (Figure S5, Panel A in the **Online Supplementary Document**). In GOLD B patients, both PD (HR = 1.489; 95% CI = 1.359–1.63) and CHF (HR = 1.384; 95% CI = 1.214–1.585) showed substantial associations with exacerbation (Figure S5, Panel B in the **Online Supplementary Document**). For GOLD E patients, PD demonstrated high risk (HR = 1.552; 95% CI = 1.411–1.708) (Figure S5, Panel C in the **Online Supplementary Document**). According to these findings, comorbidities demonstrated significant associations with moderate-to-severe COPD exacerbations. The analyses identified PD as a crucial risk factor across different GOLD groups, with HRs consistently above 1.3. Additionally, AMI and CHF presented notable risks for exacerbation. The magnitude of these associations varied among GOLD classifications, suggesting that the impact of comorbidities on COPD exacerbations may be influenced by disease severity. These results highlight the importance of comprehensive comorbidity assessment and management in COPD patients to potentially reduce exacerbation risks.

### Sensitivity analysis

We conducted an additional analysis (Model 2) using different risk factors, including age, gender, BMI, smoking status, COPD family history, CCI, airflow limitation severity, and level of health care facilities, for adjustment as a sensitivity analysis. This alternative risk stratification approach yielded results that are consistent with our main findings. Analysis revealed elevated risk among groups, with group B demonstrating a 1.3-fold increase in five-year all-cause mortality compared to group A (HR = 1.338; 95% CI = 1.250–1.433, *P* < 0.0001). Group E exhibited an even greater risk, with a 1.7-fold increase in mortality (HR = 1.750; 95% CI = 1.626–1.884, *P* < 0.0001). Similar patterns emerged for moderate-to-severe exacerbations, where group B showed a 1.3-fold higher risk (HR = 1.282; 95% CI = 1.199–1.382, *P* < 0.0001) and group E demonstrated approximately 2-fold higher risk (HR = 1.921; 95% CI = 1.782–2.071, *P* < 0.0001) compared to group A (Table S1 in the **Online Supplementary Document**).

## DISCUSSION

This comprehensive analysis of 38 928 COPD patients categorised by GOLD 2023 classification demonstrated significant differences in clinical outcomes and mortality risks across groups, with group E consistently showing the poorest prognosis. The five-year mortality rates exhibited a clear gradient, ranging from 8.4% in group A to 22.6% in group E, with HRs of 1.3 and 1.7 times higher in groups B and E compared to group A. Notably, the study suggested that comorbidity patterns were associated with clinical outcomes, where patients with cancer/metastasis and congestive heart failure appeared to have higher mortality risks across GOLD groups. The analysis also highlighted the substantial impact of pulmonary disease on moderate-to-severe exacerbations (HR = 1.491), particularly pronounced in group E patients (HR = 1.552). These findings underscore the critical importance of both GOLD classification and comorbidity assessment in determining prognosis and managing COPD patients.

Compared to real-world cohorts from other Asian countries like Korea and China, Taiwanese COPD patients in this study were older with lower CAT scores, but had a similar male proportion, BMI, smoking prevalence, and airway obstruction severity [16,42]. In contrast, the German cohort had younger patients, a more balanced gender distribution, higher BMI, and higher CAT scores, while airway obstruction severity and smoking prevalence were similar to the Taiwanese group [43]. These findings highlight unique characteristics of Taiwanese COPD patients, particularly their older age and lower CAT scores. These variations underscore the importance of accounting for regional differences in COPD management. To date, few studies have compared baseline characteristics across different GOLD 2023 classifications. The GOLD E group in Taiwan showed the highest CAT scores, severe airway obstruction, and the highest all-cause mortality, consistent with similar findings from China [42].

The primary purpose of the GOLD classification was initially designed to assist in selecting appropriate pharmacological treatments. However, in practice, it is also used to explore and evaluate the long-term disease course or prognosis of patients [44,45]. For example, Gedebjerg et al. analysed a large cohort of 33 765 COPD outpatients in Danish hospitals and found that GOLD 2017 was no better than GOLD 2011 in predicting all-cause mortality [15]. Similarly, Cabrera-Lopez et al. demonstrated in the BODE cohort that the GOLD 2017 classification was less accurate at predicting mortality compared to GOLD 2011 [46]. Additionally, Brat et al. conducted multicentre research in the Czech Republic, which showed that the GOLD 2023 classification was equally weak compared to previous A–D GOLD schemes [47]. Following this evidence from the aforementioned study, our findings indicated that the prediction of all-cause mortality was comparable between the GOLD 2017 and 2023 criteria. Despite combining groups C and D into group E, our findings found that group E is the highest risk of all-cause mortality and moderate-to-severe exacerbation, followed by group B and group A. These results were consistent with data retrieved from the cohort study Real World Research of Diagnosis and Treatment of COPD in China [42]. Here are two key aspects related to mortality prediction that merit discussion. First, we found that groups B and C showed similar outcomes, with nearly overlapping curves in the Kaplan-Meier analysis of all-cause mortality, which is similar to the result from the ‘Evaluation of COPD Longitudinally to Identify Surrogate Endpoints (ECLIPSE)’ cohort [48]. That indicates that the risk of mortality in group C is more similar to group B rather than group D. Second, the combination of group C and group D into group E, and using group A as the reference group, the mortality risk for group E falls between the original group D and group C. This suggests that merging group C and group D may introduce a potential bias in predicting mortality risk. This could be due to the exclusion of symptom factors affecting disease prognosis when evaluating under group E. This also reasonably explains the inferior predictive ability for mortality in GOLD 2023 compared to GOLD 2017. Aforementioned, patients in the GOLD E group, defined solely by their history of exacerbations, represent a high-risk population. Their frequent exacerbations and severe disease burden contribute to poor outcomes, including increased mortality. These findings emphasize the need for targeted interventions and proactive management for GOLD E patients to reduce exacerbation rates and improve prognosis, as their categorisation may overlook the impact of daily symptoms on overall health.

Moreover, GOLD 2023 group B patients’ mortality risk is similar to that of the previous group C, which may be associated with cardiovascular disease. The Danish study specifically noted that group B patients had a three-year cardiovascular mortality rate of 2.9%, significantly higher than group C’s 0.5% [49]. Despite having relatively better lung function, group B patients carried a higher cardiovascular disease burden, used more cardiovascular medications, and exhibited significantly higher cardiovascular mortality rates than group C [50]. These findings not only emphasise the importance of cardiovascular comorbidity management in COPD patients but also highlight the core value of symptom assessment in COPD clinical practice. Clinicians are advised to closely monitor potential cardiovascular risks in group B patients and implement appropriate treatment strategies.

A key finding in our study was the confirmed adherence to the proportional hazards assumption across GOLD risk groups. Our validation through log-log survival curve analysis showed parallel patterns, demonstrating that hazard ratios remained constant over time (Figure S3 in the **Online Supplementary Document**). Adjustment for entry time as a covariate produced results consistent with our primary analysis, confirming our statistical approach was appropriate (Table S2 in the **Online Supplementary Document**). Clinically, this finding is significant as it indicates the GOLD risk classification's impact on patient prognosis remains stable over time. This stability enhances the classification's value as a reliable prognostic indicator, allowing clinicians to confidently use it for risk stratification and personalised treatment decisions, ultimately improving management and quality of life for COPD patients.

COPD extends beyond being a respiratory condition, as it is frequently accompanied by numerous systemic co-morbidities that profoundly affect disease progression, treatment outcomes, and overall quality of life. Therefore, understanding the prevalence of co-morbidities helps to provide holistic patient management. This study demonstrates a significant burden of mild to moderate comorbidities across all COPD groups. Notably, the proportion of patients with severe comorbidities (≥4) was highest in group E (15.6%) and lowest in group A (12.4%), indicating that patients in group E are more prone to multiple serious comorbidities compared to those in group A. To the best of our knowledge, no directly comparable studies exist that assess comorbidity prevalence in this manner. Nevertheless, prior research has indicated that patients categorised as high-risk (GOLD groups C and D) generally have higher modified CCI scores than those in low-risk groups (GOLD groups A and B), highlighting a greater burden of comorbidities among high-risk individuals [51]. This variability underscores the importance of considering comorbidity profiles in the management of COPD patients.

COPD is associated with a high prevalence of various comorbidities, including cardiovascular diseases, hypertension, diabetes, and psychiatric disorders. In our study cohort, the most frequent prevalence of co-morbidities was PD, PUD, DM, CHF, and RED. In the Korean study, the comorbidities were defined by existing literature to evaluate the distribution of comorbidities. The most comorbidities in the Korean cohort were hypertension, asthma, diabetes, dyslipidaemia, and gastro-oesophageal reflux disease [52]. In German, hypertension, anxiety/depression, diabetes, and congestive heart failure were the most frequent co-morbidities in patients with COPD [53]. In a systematic review, which included various countries worldwide, it was indicated that comorbidities, particularly cardiovascular diseases and diabetes, are common in COPD patients [54]. These findings suggest that the prevalence and distribution of comorbidities in COPD patients vary across different regions and populations, influenced by factors such as age, gender, COPD severity, and geographical region [55,56]. Moreover, evidence on the prevalence of multi-morbidities in COPD is limited. Our findings reveal that the network of co-occurring chronic diseases is highly complex. In the Taiwanese COPD cohort, the most frequent co-occurrences across all GOLD stages were pulmonary disease with CHF, PUD, and DM. Previous studies have shown that the coexistence of these conditions increases the risk of exacerbations and mortality [57–59], emphasising the need for tailored public health strategies to effectively manage COPD and its associated comorbidities through a patient-centred rather than a single-disease approach.

COPD is associated with a higher risk of various cardiovascular diseases, including myocardial infarction, ischemic heart disease, heart failure, and arrhythmias [60]. The risk of sudden cardiac death is notably higher in COPD patients, especially those with frequent exacerbations [3]. Moreover, patients with COPD face an increased risk of cardiovascular disease (CVD) and associated mortality. This relationship is influenced by various factors, including acute exacerbations of COPD and the presence of cardiovascular comorbidities [61,62]. Similar to previous studies, our findings demonstrated that cardiovascular comorbidities, AMI, CHF, and PVD increased all-cause mortality and moderate-to-severe exacerbation risks across all GOLD categories. Despite the high risk, CVD in COPD patients is often under-recognised and undertreated, highlighting the need for better clinical guidelines and proactive management strategies [63]. The interplay between COPD and cardiovascular events underscores the importance of integrated care approaches to manage both conditions effectively.

Our study has significant implications for global COPD management by validating the GOLD 2023 classification system in a large Asian population, providing evidence that this framework effectively stratifies mortality risk and clinical outcomes across different patient groups. The observed clear mortality gradient (from 8.4% in group A to 22.6% in group E) suggests that treatment intensification should be prioritised for patients in groups B and E, particularly those with comorbid cancer/metastasis and congestive heart failure. We recommend implementing routine comprehensive comorbidity screening for all COPD patients and developing personalised management approaches based on specific comorbidity profiles. Patients with cardiovascular comorbidities should receive optimised cardiopulmonary care and closer monitoring, as suggested by the integrated care model in the Spanish study [64]; while patients with malignancies require integrated oncology-pulmonology care pathways. Future studies should focus on validating these findings in diverse global populations, particularly in resource-limited settings where comorbidity patterns may differ. Prospective multicentre trials investigating targeted interventions based on GOLD classification and comorbidity profiles are needed, alongside studies examining the impact of comorbidity-specific interventions on exacerbation rates and mortality. We utilised a large registry-based Asian cohort to comprehensively evaluate the GOLD 2023 classification system, providing valuable complementary perspectives for research in COPD, comorbidities, and their prognosis. This work contributes to enhancing our understanding of COPD disease characteristics across different populations and provides clinicians with stronger evidence to improve patient care and prognostic assessment, ultimately advancing the optimisation and development of global COPD management strategies.

We identified several key risk factors for mortality and exacerbation in COPD patients, including advanced age, smoking history, lower BMI, severe airflow obstruction, presence of wheezing, and higher CCI, supporting previous findings [65–68]. Notably, wheezing emerged as an independent risk factor not only for moderate-to-severe exacerbations but also for all-cause mortality, highlighting its critical role as an important clinical phenotype in COPD as a previous study reported [65]. Given the association between wheezing and frequent exacerbations, and greater impairment in lung function, wheezing may serve as a valuable and intuitive marker for assessing symptom severity in COPD in clinical practice. However, evidence regarding the association between wheezing and all-cause mortality in COPD is limited. In a previous study, the participants with ACO exhibited a significant increase in all-cause mortality compared to those without [66]. One of the possible explanations of our finding is that patients with wheezing in group E have a higher proportion of patients with coexistence of asthma, which contributes to the higher risk of all-cause death.

At baseline, it was observed that 28.3% of group A patients were using ICS-containing inhalers, a finding worth discussing, as this data reflects medication usage measured during the first baseline assessment when patients initially entered Taiwan's COPD P4P program. This relatively high percentage directly demonstrates the gap between routine clinical practice and clinical guidelines (which discourage ICS use in less symptomatic patients). Factors contributing to this discrepancy may include: physicians' reluctance to discontinue established treatment regimens even after patient symptom improvement, and patients' resistance to medication adjustments due to psychological reassurance or perceived efficacy [69,70]. Taiwan's COPD P4P program addresses this issue through its standardised care model, which requires all participants to undergo a comprehensive reassessment of disease status after entering the program, followed by appropriate medication adjustments. This systematic intervention helps identify and correct previously inappropriate medication use, including unnecessary ICS use among group A patients. Such programs are crucial for narrowing the gap between guidelines and practice, not only improving treatment appropriateness but also reducing unnecessary resource consumption and minimising potential side effects.

There are some limitations to this study. First, our study cohort is not population-based, which may limit the generalizability of results. Significant differences exist between the P4P and non-P4P groups had been reported, including slightly older, having lower CCI scores, lower single-inhaler but higher dual-inhaler usage rates, and better medication adherence [71], suggesting that P4P program patients may have better treatment compliance due to more regular follow-ups or improved health care access. Despite this selection bias, our patients were recruited from multiple health care facilities of varying levels, and stratified analysis by health care facility level showed consistent results, demonstrating the robustness of our conclusions (Table S3 in the **Online Supplementary Document**). Second, despite accounting for various potential confounders in this real-world study, we acknowledge limitations including residual confounding factors (environmental exposures, physical activity, and nutrition) and the absence of important COPD prognostic factors such as genetic backgrounds in our database. Additionally, as our study focused on a Taiwanese cohort, results may have limited generalisability to other populations with different health care systems or genetic profiles. Future research should incorporate these variables and extend findings across diverse populations and health care contexts. Third, some degree of recall bias may exist in our collection of self-reported smoking history and lifestyle factors. To minimise this potential bias, all health care professionals involved in data collection underwent professional training to ensure standardised interview processes. We implemented validated structured questionnaires for follow-up care, reducing inconsistencies in question formulation. During statistical analysis, we adjusted for relevant confounding factors to mitigate this bias. For future research, we recommend incorporating objective measurement methods, such as biomarker detection (for example, fractional exhaled nitric oxide, which serves as a useful indicator for determining smoking intensity), to enhance data reliability. Fourth, underreporting or misclassification of comorbidities was possible. To minimise this bias, we defined comorbidities as present if the relevant code appeared at least once in admission records or three times in outpatient department diagnosis codes. The validity of this method has been proven [72]. Moreover, we applied the well-validated ICD coding algorithm based on the CDMF CCI scheme described by Glasheen et al. This approach provides consistency in condition identification and has demonstrated reliability across multiple populations. By adopting this validated coding system, we were able to mitigate the potential impact of data quality issues on our research results [39]. Fifth, the potential limitation of using ICD-10 codes and claims-based definitions for identifying outcomes, as this approach may introduce some degree of outcome misclassification. Although our definitions for outcomes are consistent with methodologies employed in previous research, we recognize that claims data cannot perfectly capture all clinical nuances. Nevertheless, we believe that any potential misclassification would occur randomly across our three groups, thereby preserving the validity of our between-group comparisons and the overall reliability of our findings. Sixth, this study is the lack of cause-of-death information in our database, which prevented us from distinguishing between respiratory-related deaths and deaths from other causes. This may have resulted in non-respiratory deaths obscuring the accurate assessment of COPD-specific mortality. Therefore, our all-cause mortality analysis results should be interpreted with caution. Future studies with access to cause-specific mortality data would help more precisely elucidate the associations between predictors and COPD-specific outcomes.

## CONCLUSIONS

In conclusion, in this study, we reinforced the clinical value of the GOLD 2023 classification system for risk stratification in COPD patients. The notable association between specific comorbidities and adverse outcomes underscores the critical need for comprehensive patient assessment beyond conventional COPD parameters. The consistent impact of cardiovascular and pulmonary comorbidities across all GOLD groups suggests that systematic comorbidity screening and management should be integrated into routine COPD care protocols. These findings provide clinicians with valuable insights for developing more targeted therapeutic approaches and emphasize the importance of personalized treatment strategies that consider both COPD severity and comorbidity burden to optimize patient outcomes.

## Additional material


Online Supplementary Document

## References

[1] Safiri S Carson-Chahhoud K Noori M Nejadghaderi SA Sullman MJM Heris JA Burden of chronic obstructive pulmonary disease and its attributable risk factors in 204 countries and territories, 1990- 2019: results from the global burden of disease study 2019. BMJ. 2022;378:e069679. 10.1136/bmj-2021-069679 35896191 PMC9326843

[2] Chen S Kuhn M Prettner K Yu F Yang T Bärnighausen T The global economic burden of chronic obstructive pulmonary disease for 204 countries and territories in 2020-50: a health-augmented macroeconomic modelling study. Lancet Glob Health. 2023;11:e1183–93. 10.1016/S2214-109X(23)00217-6 37474226 PMC10369014

[3] Boers E Barrett M Su JG Benjafield AV Sinha S Kaye L Global Burden of Chronic Obstructive Pulmonary Disease Through 2050. JAMA Netw Open. 2023;6:e2346598. 10.1001/jamanetworkopen.2023.46598 38060225 PMC10704283

[4] Celli B Fabbri L Criner G Martinez FJ Mannino D Vogelmeier C Definition and nomenclature of chronic obstructive pulmonary disease: time for its revision. Am J Respir Crit Care Med. 2022;206:1317–25. 10.1164/rccm.202204-0671PP 35914087 PMC9746870

[5] Venkatesan P GOLD COPD report: 2023 update. Lancet Respir Med. 2023;11:18. 10.1016/S2213-2600(22)00494-5 36462509

[6] Skajaa N Laugesen K Horváth-Puhó E Sørensen HT Comorbidities and mortality among patients with chronic obstructive pulmonary disease. BMJ Open Respir Res. 2023;10:e001798. 10.1136/bmjresp-2023-001798 37797964 PMC10551998

[7] Fabbri LM Celli BR Agustí A Criner GJ Dransfield MT Divo M COPD and multimorbidity: recognising and addressing a syndemic occurrence. Lancet Respir Med. 2023;11:1020–34. 10.1016/S2213-2600(23)00261-8 37696283

[8] Global Strategy for the Diagnosis, Management and Prevention of COPD, Global Initiative for Chronic Obstructive Lung Disease (GOLD). Global Initiative for Chronic Lung Disease (2023 Report). Fontana, Wisconsin, USA: Global Initiative for Chronic Lung Disease; 2023. Available: https://goldcopd.org/2023-gold-report-2/. Accessed: 30 December 2024.

[9] Tudoric N Koblizek V Miravitlles M Valipour A Milenkovic B Barczyk A GOLD 2017 on the way to a phenotypic approach? Analysis from the Phenotypes of COPD in Central and Eastern Europe (POPE) Cohort. Eur Respir J. 2017;49:1602518. 10.1183/13993003.02518-2016 28446560

[10] Haughney J Gruffydd-Jones K Roberts J Lee AJ Hardwell A McGarvey L The distribution of COPD in UK general practice using the new GOLD classification. Eur Respir J. 2014;43:993–1002. 10.1183/09031936.00065013 24176990

[11] Çolak Y Afzal S Marott JL Nordestgaard BG Vestbo J Ingebrigtsen TS Prognosis of COPD depends on severity of exacerbation history: A population-based analysis. Respir Med. 2019;155:141–7. 10.1016/j.rmed.2019.07.021 31362177

[12] Vogelmeier CF Friedrich FW Timpel P Kossack N Diesing J Pignot M Impact of COPD on mortality: An 8-year observational retrospective healthcare claims database cohort study. Respir Med. 2024;222:107506. 10.1016/j.rmed.2023.107506 38151176

[13] Miravitlles M Kostikas K Bizymi N Tzanakis N A Novel Figure and Algorithm for the Gold ABE Classification. Arch Bronconeumol. 2023;59:702–4. 10.1016/j.arbres.2023.06.001 37355409

[14] Criner RN Labaki WW Regan EA Bon JM Soler X Bhatt SP Mortality and Exacerbations by Global Initiative for Chronic Obstructive Lung Disease Groups ABCD: 2011 Versus 2017 in the COPDGene® Cohort. Chronic Obstr Pulm Dis (Miami). 2019;6:64–73. 10.15326/jcopdf.6.1.2018.0130 30775425 PMC6373581

[15] Gedebjerg A Szépligeti SK Wackerhausen LH Horváth-Puhó E Dahl R Hansen JG Prediction of mortality in patients with chronic obstructive pulmonary disease with the new Global Initiative for Chronic Obstructive Lung Disease 2017 classification: a cohort study. Lancet Respir Med. 2018;6:204–12. 10.1016/S2213-2600(18)30002-X 29331311

[16] Song JH Lee CH Um SJ Park YB Yoo KH Jung KS Clinical impacts of the classification by 2017 GOLD guideline comparing previous ones on outcomes of COPD in real-world cohorts. Int J Chron Obstruct Pulmon Dis. 2018;13:3473–84. 10.2147/COPD.S177238 30498337 PMC6207379

[17] Cheng W Zhou A Zeng Y Lin L Song Q Liu C Prediction of Hospitalization and Mortality in Patients with Chronic Obstructive Pulmonary Disease with the New Global Initiative for Chronic Obstructive Lung Disease 2023 Group Classification: A Prospective Cohort and a Retrospective Analysis. Int J Chron Obstruct Pulmon Dis. 2023;18:2341–52. 10.2147/COPD.S429104 37908629 PMC10615105

[18] Westerik JA Metting EI van Boven JF Tiersma W Kocks JW Schermer TR Associations between chronic comorbidity and exacerbation risk in primary care patients with COPD. Respir Res. 2017;18:31. 10.1186/s12931-017-0512-2 28166777 PMC5294875

[19] Almagro P Cabrera FJ Diez J Boixeda R Alonso Ortiz MB Murio C Comorbidities and short-term prognosis in patients hospitalized for acute exacerbation of COPD: the EPOC en Servicios de medicina interna (ESMI) study. Chest. 2012;142:1126–33. 10.1378/chest.11-2413 23303399

[20] Argano C Scichilone N Natoli G Nobili A Corazza GR Mannucci PM Pattern of comorbidities and 1-year mortality in elderly patients with COPD hospitalized in internal medicine wards: data from the RePoSI Registry. Intern Emerg Med. 2021;16:389–400. 10.1007/s11739-020-02412-1 32720248 PMC7384278

[21] de Torres JP Casanova C Marín JM Pinto-Plata V Divo M Zulueta JJ Prognostic evaluation of COPD patients: GOLD 2011 versus BODE and the COPD comorbidity index COTE. Thorax. 2014;69:799–804. 10.1136/thoraxjnl-2014-205770 24969641

[22] Li CL Lin MH Chen PS Tsai YC Shen LS Kuo HC Using the BODE Index and Comorbidities to Predict Health Utilization Resources in Chronic Obstructive Pulmonary Disease. Int J Chron Obstruct Pulmon Dis. 2020;15:389–95. 10.2147/COPD.S234363 32110007 PMC7036670

[23] Santos NCD Miravitlles M Camelier AA Almeida VDC Maciel RRBT Camelier FWR Prevalence and Impact of Comorbidities in Individuals with Chronic Obstructive Pulmonary Disease: A Systematic Review. Tuberc Respir Dis (Seoul). 2022;85:205–20. 10.4046/trd.2021.0179 35618259 PMC9263346

[24] Lin YY Huang CS Aging in Taiwan: Building a Society for Active Aging and Aging in Place. Gerontologist. 2016;56:176–83. 10.1093/geront/gnv107 26589450

[25] Antoniou ASG, Burke RJ, Cooper CL. The aging workforce handbook: individual, organizational and societal challenges. 1st ed. Bingley, UK: Emerald Publishing Limited; 2016.

[26] Wing K Williamson E Carpenter JR Wise L Schneeweiss S Smeeth L Real-world effects of medications for chronic obstructive pulmonary disease: protocol for a UK population-based non-interventional cohort study with validation against randomised trial results. BMJ Open. 2018;8:e019475. 10.1136/bmjopen-2017-019475 29581202 PMC5875594

[27] Wing K Williamson E Carpenter JR Wise L Schneeweiss S Smeeth L Real world effects of COPD medications: a cohort study with validation against results from randomised controlled trials. Eur Respir J. 2021;57:2001586. 10.1183/13993003.01586-2020 33093119 PMC8176192

[28] Burns L Roux NL Kalesnik-Orszulak R Christian J Hukkelhoven M Rockhold F Real-World Evidence for Regulatory Decision-Making: Guidance From Around the World. Clin Ther. 2022;44:420–37. 10.1016/j.clinthera.2022.01.012 35181179

[29] Trotter JP Patient registries: a new gold standard for “real world” research. Ochsner J. 2002;4:211–4. 22826660 PMC3400525

[30] Sedrakyan A Marinac-Dabic D Campbell B Aryal S Baird CE Goodney P Advancing the Real-World Evidence for Medical Devices through Coordinated Registry Networks. BMJ Surg Interv Health Technol. 2022;4:e000123. 10.1136/bmjsit-2021-000123 36393894 PMC9660584

[31] Gliklich RE Leavy MB Assessing Real-World Data Quality: The Application of Patient Registry Quality Criteria to Real-World Data and Real-World Evidence. Ther Innov Regul Sci. 2020;54:303–7. 10.1007/s43441-019-00058-6 32072577

[32] Ip MS Ko FW Lau AC Yu WC Tang KS Choo K Updated spirometric reference values for adult Chinese in Hong Kong and implications on clinical utilization. Chest. 2006;129:384–92. 10.1378/chest.129.2.384 16478856

[33] Eltayara L Becklake MR Volta CA Milic-Emili J Relationship between chronic dyspnea and expiratory flow limitation in patients with chronic obstructive pulmonary disease. Am J Respir Crit Care Med. 1996;154:1726–34. 10.1164/ajrccm.154.6.8970362 8970362

[34] Jones PW Harding G Berry P Wiklund I Chen WH Kline Leidy N Development and first validation of the COPD Assessment Test. Eur Respir J. 2009;34:648–54. 10.1183/09031936.00102509 19720809

[35] Chen PJ Yang KY Perng WC Lin KC Wang KY Effect of dyspnea on frailty stages and related factors in Taiwanese men with COPD. Int J Chron Obstruct Pulmon Dis. 2018;13:2463–9. 10.2147/COPD.S172694 30147312 PMC6101740

[36] Wiklund I Berry P Lu KX Fang J Fu C The Chinese translation of COPD Assessment TestTM (CAT) provides a valid and reliable measurement of COPD health status in Chinese COPD patients. Am J Respir Crit Care Med. 2010;181:3575.

[37] Cheng SL Lin CH Wang CC Chan MC Hsu JY Hang LW Comparison between COPD Assessment Test (CAT) and modified Medical Research Council (mMRC) dyspnea scores for evaluation of clinical symptoms, comorbidities and medical resources utilization in COPD patients. J Formos Med Assoc. 2019;118:429–35. 10.1016/j.jfma.2018.06.018 30150099

[38] Chen WY Hsiao CH Chen YC Ho CH Wang JJ Hsing CH Cisplatin Nephrotoxicity Might Have a Sex Difference. An analysis Based on Women’s Sex Hormone Changes. J Cancer. 2017;8:3939–44. 10.7150/jca.20083 29187868 PMC5705995

[39] Glasheen WP Cordier T Gumpina R Haugh G Davis J Renda A Charlson Comorbidity Index: ICD-9 Update and ICD-10 Translation. Am Health Drug Benefits. 2019;12:188–97. 31428236 PMC6684052

[40] Wallström O Stridsman C Lindberg A Nyberg F Vanfleteren LEGW Exacerbation History and Risk of Myocardial Infarction and Pulmonary Embolism in COPD. Chest. 2024;166:1347–59. 10.1016/j.chest.2024.07.150 39094732 PMC11638550

[41] Wu YK Su WL Yang MC Chen SY Wu CW Lan CC Characterization Associated with the Frequent Severe Exacerbator Phenotype in COPD Patients. Int J Chron Obstruct Pulmon Dis. 2021;16:2475–85. 10.2147/COPD.S317177 34511892 PMC8416186

[42] Lin L Song Q Cheng W Li T Zhang P Liu C Impact of exacerbation history on future risk and treatment outcomes in chronic obstructive pulmonary disease patients: A prospective cohort study based on Global Initiative for Chronic Obstructive Lung Disease (GOLD) A and B classifications. J Glob Health. 2024;14:04202. 10.7189/jogh.14.04202 39388682 PMC11466499

[43] Worth H Buhl R Criée CP Kardos P Mailänder C Vogelmeier C The ‘real-life’ COPD patient in Germany: The DACCORD study. Respir Med. 2016;111:64–71. 10.1016/j.rmed.2015.12.010 26775251

[44] Soriano JB Brighter than GOLD. Lancet Respir Med. 2018;6:165–6. 10.1016/S2213-2600(18)30001-8 29331312

[45] Singh D Barnes PJ Stockley R Lopez Valera MV Vogelmeier C Agusti A Pharmacological treatment of COPD: the devil is always in the detail. Eur Respir J. 2018;51:1800263. 10.1183/13993003.00263-2018 29674480

[46] Cabrera López C Casanova Macario C Marín Trigo JM de-Torres JP Sicilia Torres R González JM Comparison of the 2017 and 2015 Global Initiative for Chronic Obstructive Lung Disease Reports. Impact on Grouping and Outcomes. Am J Respir Crit Care Med. 2018;197:463–9. 10.1164/rccm.201707-1363OC 29099607

[47] Brat K Svoboda M Zatloukal J Plutinsky M Volakova E Popelkova P Prognostic Properties of the GOLD 2023 Classification System. Int J Chron Obstruct Pulmon Dis. 2023;18:661–7. 10.2147/COPD.S410372 37114105 PMC10126720

[48] Faner R Noell G Badia JR López-Giraldo A Bakke P Silverman EK Distribution, temporal stability and association with all-cause mortality of the 2017 GOLD groups in the ECLIPSE cohort. Respir Med. 2018;141:14–9. 10.1016/j.rmed.2018.06.015 30053959

[49] Lange P Marott JL Vestbo J Olsen KR Ingebrigtsen TS Dahl M Prediction of the clinical course of chronic obstructive pulmonary disease, using the new GOLD classification: a study of the general population. Am J Respir Crit Care Med. 2012;186:975–81. 10.1164/rccm.201207-1299OC 22997207

[50] Dusser D Wise RA Dahl R Anzueto A Carter K Fowler A Differences in outcomes between GOLD groups in patients with COPD in the TIOSPIR(®) trial. Int J Chron Obstruct Pulmon Dis. 2016;11:133–45. 10.2147/COPD.S97924 26855568 PMC4725639

[51] Özden Sertçelik Ü Sertçelik A Huseynova K Öncel A Damadoğlu E Çöplü L The Association Between Comorbidities and a High-Risk Status According to COPD GOLD Groups. Acta Medica (Cordoba). 2022;54:27–34. 10.32552/2022.ActaMedica.818

[52] Kim Y Kim YJ Cho WK Effect of multiple comorbidities on mortality in chronic obstructive pulmonary disease among Korean population: a nationwide cohort study. BMC Pulm Med. 2021;21:56. 10.1186/s12890-021-01424-7 33573635 PMC7879613

[53] Greulich T Weist BJD Koczulla AR Janciauskiene S Klemmer A Lux W Prevalence of comorbidities in COPD patients by disease severity in a German population. Respir Med. 2017;132:132–8. 10.1016/j.rmed.2017.10.007 29229085

[54] Santos NCD Miravitlles M Camelier AA Almeida VDC Maciel RRBT Camelier FWR Prevalence and Impact of Comorbidities in Individuals with Chronic Obstructive Pulmonary Disease: A Systematic Review. Tuberc Respir Dis (Seoul). 2022;85:205–20. 10.4046/trd.2021.0179 35618259 PMC9263346

[55] Yin HL Yin SQ Lin QY Xu Y Xu HW Liu T Prevalence of comorbidities in chronic obstructive pulmonary disease patients: A meta-analysis. Medicine (Baltimore). 2017;96:e6836. 10.1097/MD.0000000000006836 28489768 PMC5428602

[56] Nagorni-Obradovic LM Vukovic DS The prevalence of COPD co-morbidities in Serbia: results of a national survey. NPJ Prim Care Respir Med. 2014;24:14008. 10.1038/npjpcrm.2014.8 24921714 PMC4373300

[57] Axson EL Bottle A Cowie MR Quint JK Relationship between heart failure and the risk of acute exacerbation of COPD. Thorax. 2021;76:807–14. 10.1136/thoraxjnl-2020-216390 33927022 PMC8311079

[58] Figueira Gonçalves JM García Bello MÁ Golpe R Alonso Jerez JL García-Talavera I Impact of diabetes mellitus on the risk of severe exacerbation in patients with chronic obstructive pulmonary disease. Clin Respir J. 2020;14:1208–11. 10.1111/crj.13255 32781483

[59] Ho TW Huang CT Ruan SY Tsai YJ Lai F Yu CJ Diabetes mellitus in patients with chronic obstructive pulmonary disease-The impact on mortality. PLoS One. 2017;12:e0175794. 10.1371/journal.pone.0175794 28410410 PMC5391945

[60] Chen W Thomas J Sadatsafavi M FitzGerald JM Risk of cardiovascular comorbidity in patients with chronic obstructive pulmonary disease: a systematic review and meta-analysis. Lancet Respir Med. 2015;3:631–9. 10.1016/S2213-2600(15)00241-6 26208998

[61] Rothnie KJ Yan R Smeeth L Quint JK Risk of myocardial infarction (MI) and death following MI in people with chronic obstructive pulmonary disease (COPD): a systematic review and meta-analysis. BMJ Open. 2015;5:e007824. 10.1136/bmjopen-2015-007824 26362660 PMC4567661

[62] Wang M Lin EP Huang LC Li CY Shyr Y Lai CH Mortality of Cardiovascular Events in Patients With COPD and Preceding Hospitalization for Acute Exacerbation. Chest. 2020;158:973–85. 10.1016/j.chest.2020.02.046 32184108

[63] Morgan AD Zakeri R Quint JK Defining the relationship between COPD and CVD: what are the implications for clinical practice? Ther Adv Respir Dis. 2018;12:1753465817750524. 10.1177/1753465817750524 29355081 PMC5937157

[64] de Miguel-Díez J Núñez Villota J Santos Pérez S Manito Lorite N Alcázar Navarrete B Delgado Jiménez JF Multidisciplinary Management of Patients With Chronic Obstructive Pulmonary Disease and Cardiovascular Disease. Arch Bronconeumol. 2024;60:226–37. 10.1016/j.arbres.2024.01.013 38383272

[65] Seo H Kim Y Jang JG Ahn JH Ra SW Park YB Clinical implications of wheezing in patients with chronic obstructive pulmonary disease. J Thorac Dis. 2023;15:6047–57. 10.21037/jtd-23-1031 38090295 PMC10713320

[66] Zhu M Chen A Epidemiological characteristics of asthma-COPD overlap, its association with all-cause mortality, and the mediating role of depressive symptoms: evidence from NHANES 2005-2018. BMC Public Health. 2024;24:1423. 10.1186/s12889-024-18911-1 38807148 PMC11134654

[67] Müllerova H Maselli DJ Locantore N Vestbo J Hurst JR Wedzicha JA Hospitalized exacerbations of COPD: risk factors and outcomes in the ECLIPSE cohort. Chest. 2015;147:999–1007. 10.1378/chest.14-0655 25356881

[68] Berry CE Wise RA Mortality in COPD: causes, risk factors, and prevention. COPD. 2010;7:375–82. 10.3109/15412555.2010.510160 20854053 PMC7273182

[69] Gilworth G Harries T Corrigan C Thomas M White P Perceptions of COPD patients of the proposed withdrawal of inhaled corticosteroids prescribed outside guidelines: A qualitative study. Chron Respir Dis. 2019;16:1479973119855880. 10.1177/1479973119855880 31195812 PMC6566471

[70] Chalmers JD Miravitlles M Withdrawal of inhaled corticosteroids in COPD. Eur Respir J. 2020;56:2001778. 10.1183/13993003.01778-2020 32646895

[71] Cheng SL Li YR Huang N Yu CJ Wang HC Lin MC Effectiveness of Nationwide COPD Pay-for-Performance Program on COPD Exacerbations in Taiwan. Int J Chron Obstruct Pulmon Dis. 2021;16:2869–81. 10.2147/COPD.S329454 34703221 PMC8539057

[72] Chen WY Hsiao CH Chen YC Ho CH Wang JJ Hsing CH Cisplatin Nephrotoxicity Might Have a Sex Difference. An analysis Based on Women’s Sex Hormone Changes. J Cancer. 2017;8:3939–44. 10.7150/jca.20083 29187868 PMC5705995

