# Supplementary material for: Prognostic risk profiling in COPD using Global Initiative for Chronic Obstructive Lung Disease 2023 ABE and comorbidity assessment: evidence from a register-based COPD cohort
Source: J Glob Health. 2025 May 23;15:04152. doi: 10.7189/jogh.15.04152 (PMC12100575; doi:10.7189/jogh.15.04152)
Supplement: Online Supplementary Document [file jogh-15-04152-s001.pdf]

Supplement to: Lin CH, Li YR, Cheng SL, Wang HC, Lin HI, Lee KY, Chong IW, Chan PC, Chen HW, Yu CJ. Prognostic risk profiling in COPD using Global Initiative for Chronic Obstructive Lung Disease 2023 ABE and comorbidity assessment: evidence from a register-based COPD. J Glob Health. 2025;15:04152.

Supplemental Figure 1. Transition from GOLD 2017 to GOLD 2023 Classification

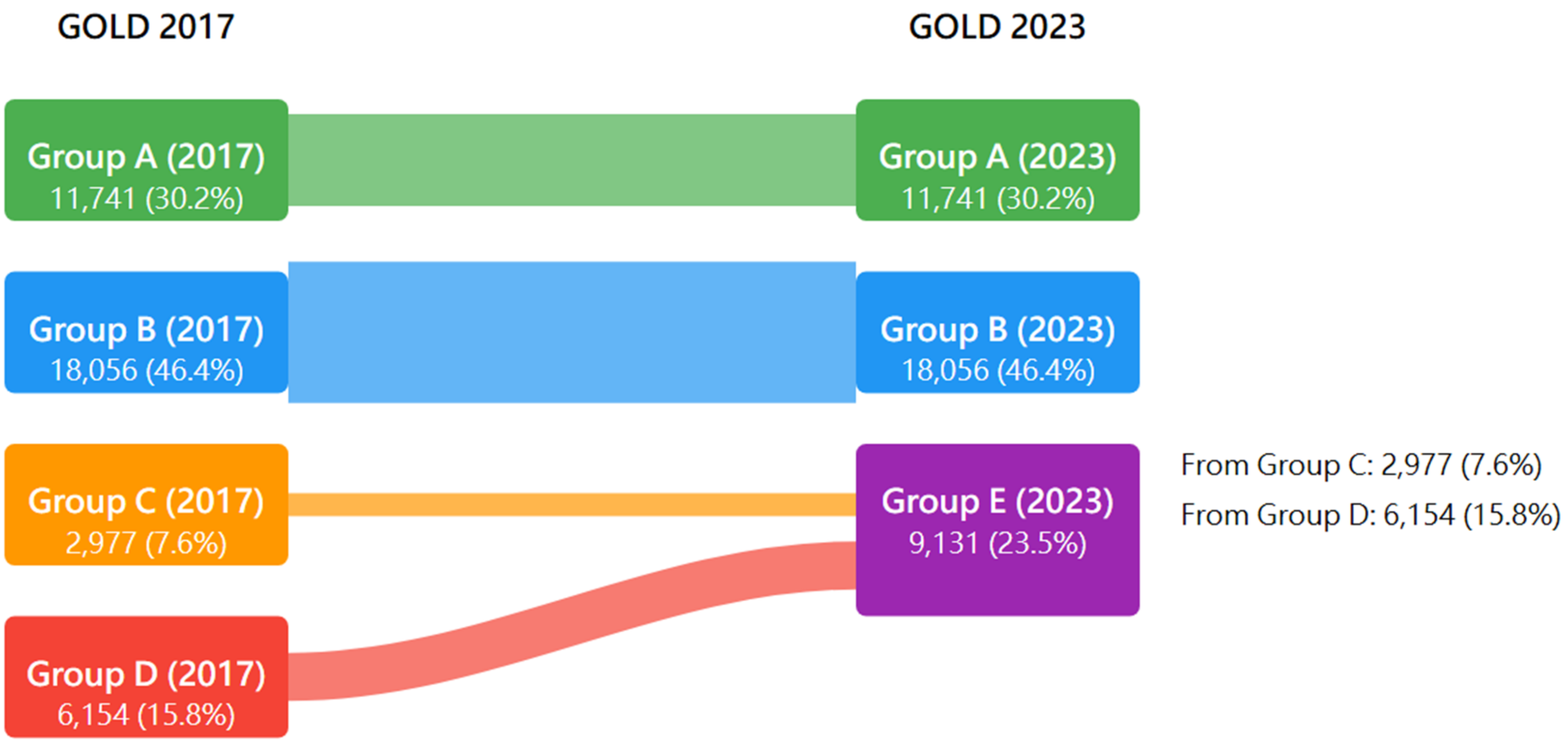

Supplemental Figure 2. Risk factor of all-cause mortality and moderate-to-severe exacerbation across GOLD 2023 classification

(A) All-cause mortality

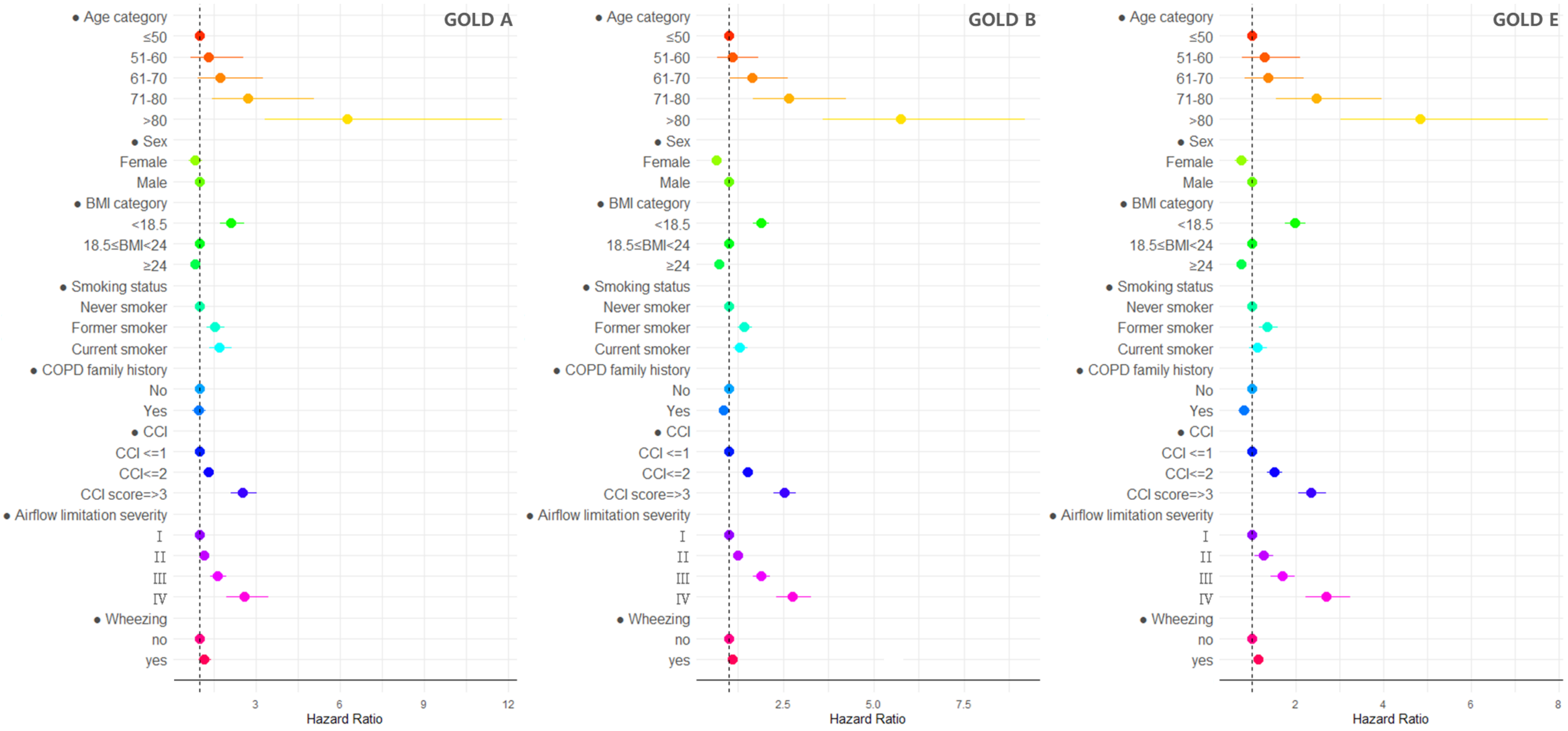

(B) moderate-to-severe exacerbation

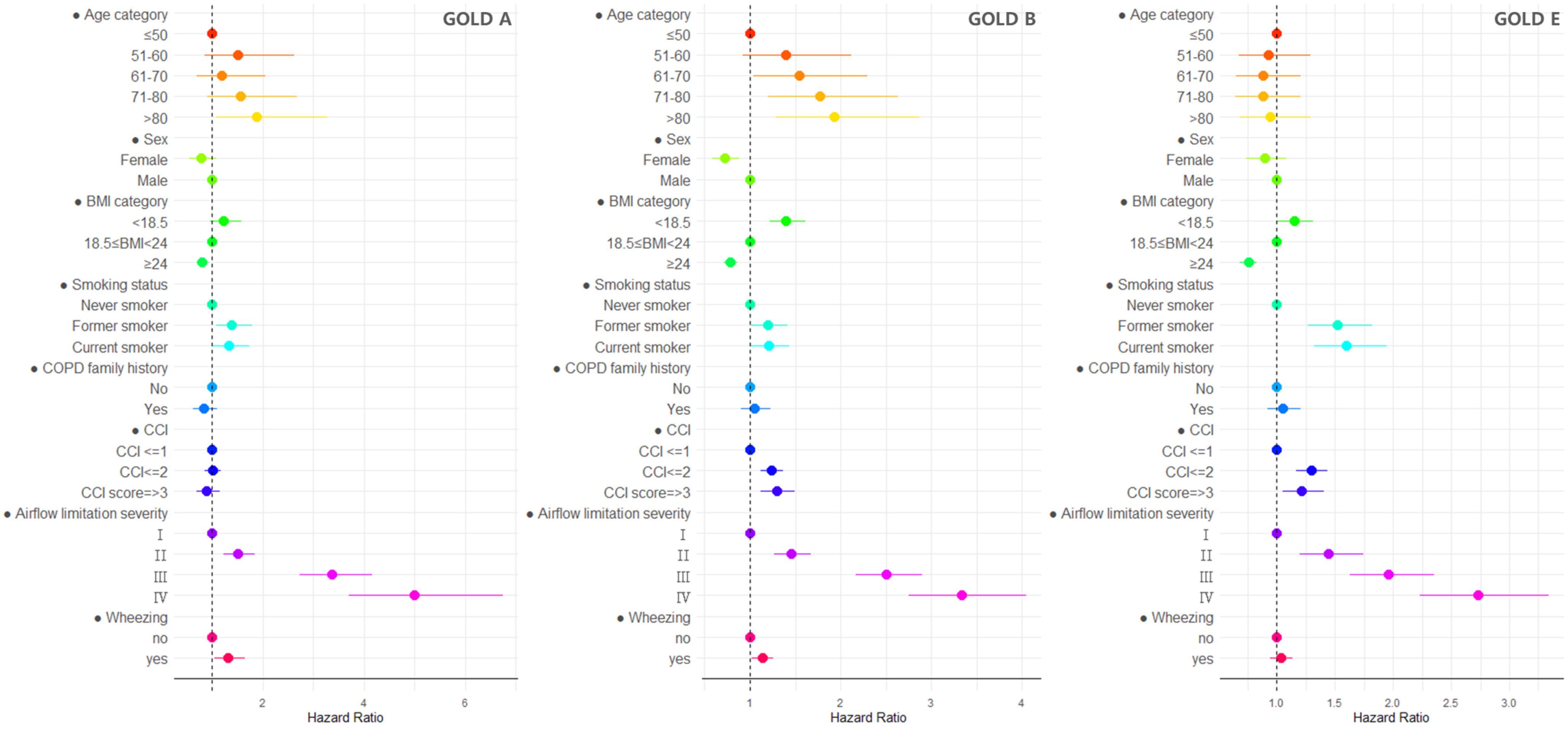

Supplemental Figure 3. Graphical analysis of log-log survival curves across GOLD 2023 classification

(A) log-log survival curves of mortality

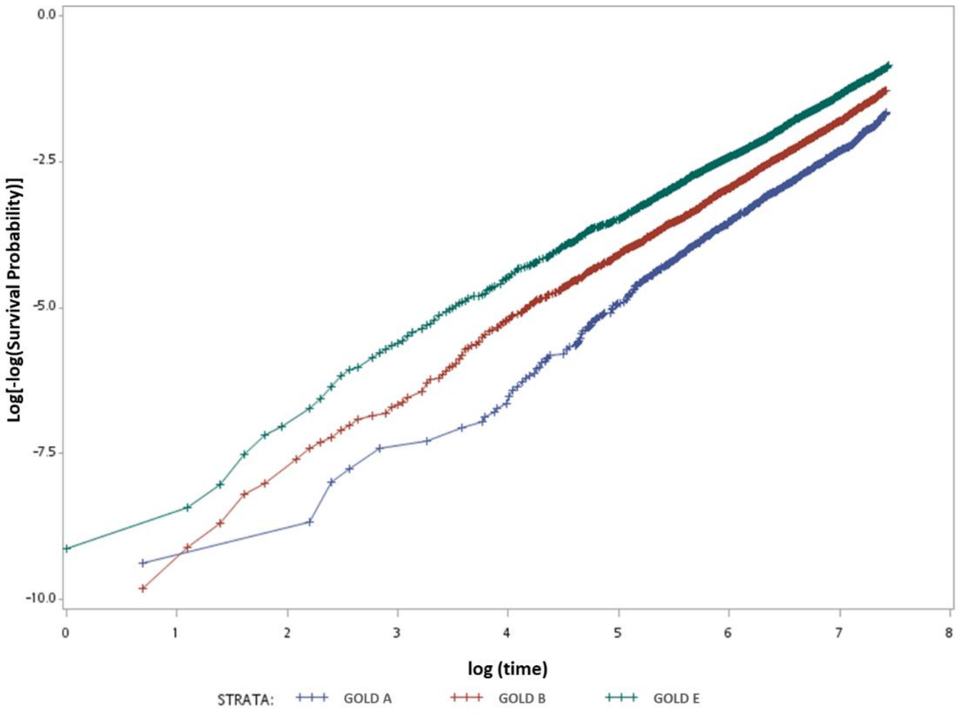

(B) log-log survival curves of moderate-to-severe acute exacerbation

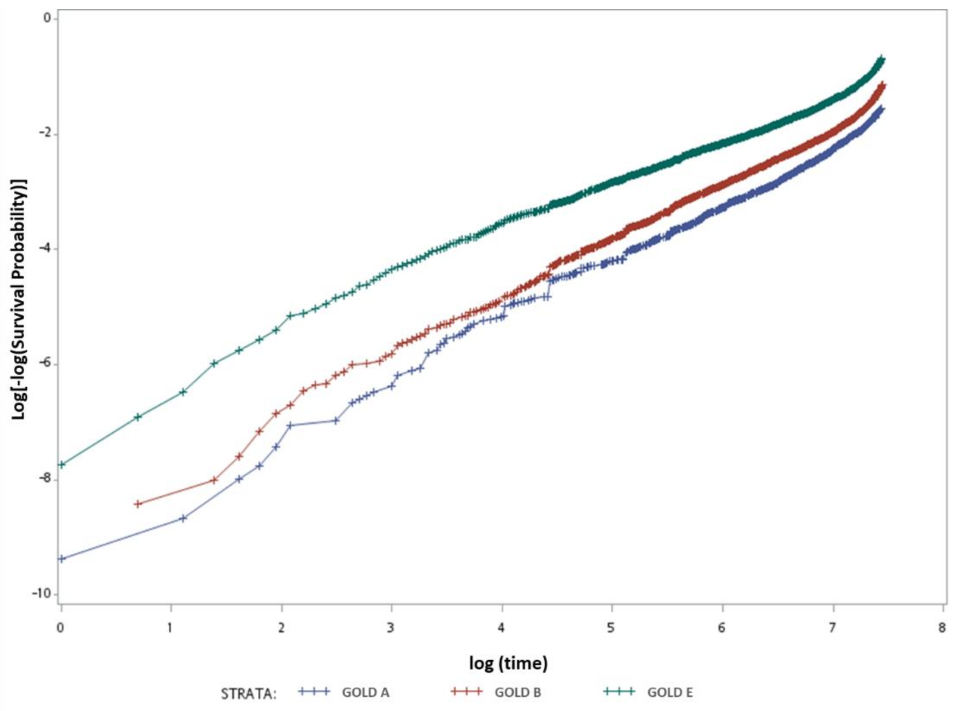

Supplemental Figure 4. Forest plot of the relationship between comorbidities and all-cause mortality in patients with COPD across GOLD 2023 classification

(A) GOLD A (B) GOLD B (C) GOLD E

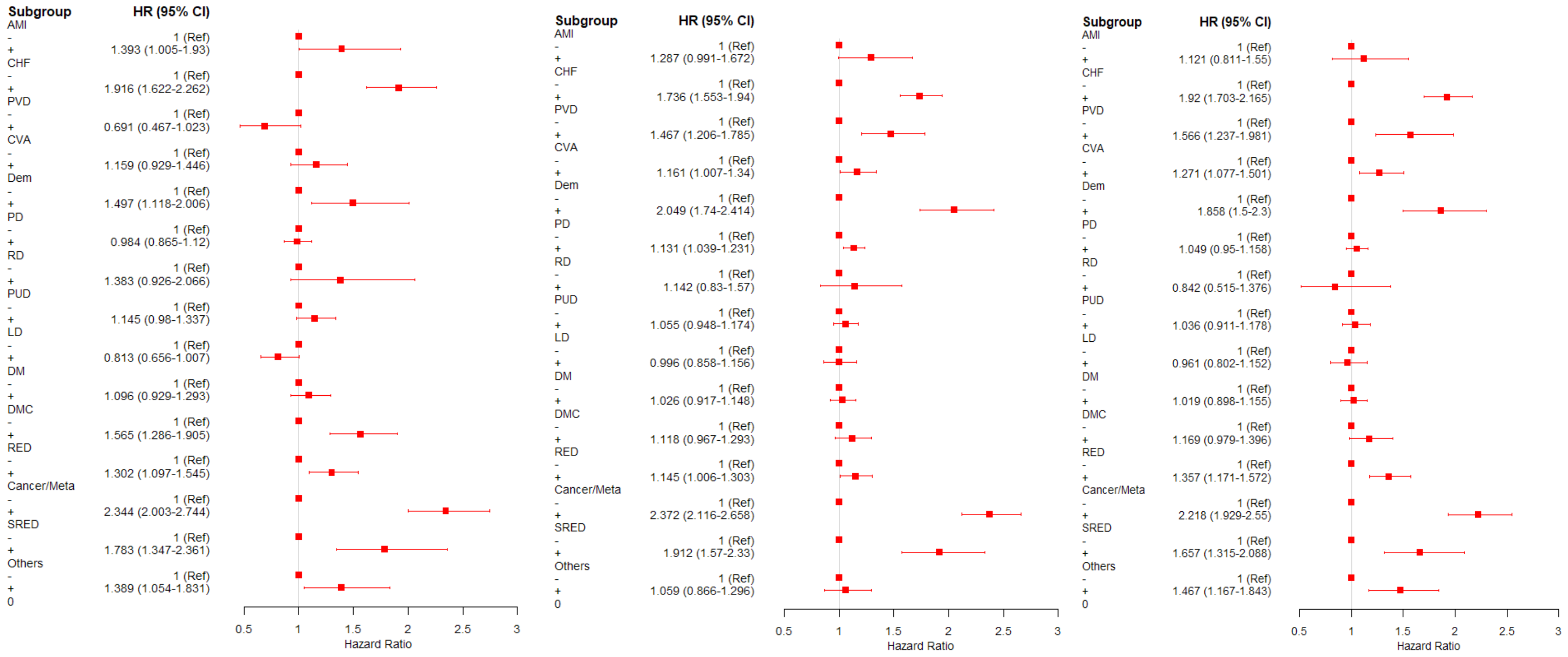

Supplemental Figure 5. Forest plot of the relationship between comorbidities and moderate-to-severe exacerbation in patients with COPD across GOLD 2023 classification

(A) GOLD A

(B) GOLD B

(C) GOLD E

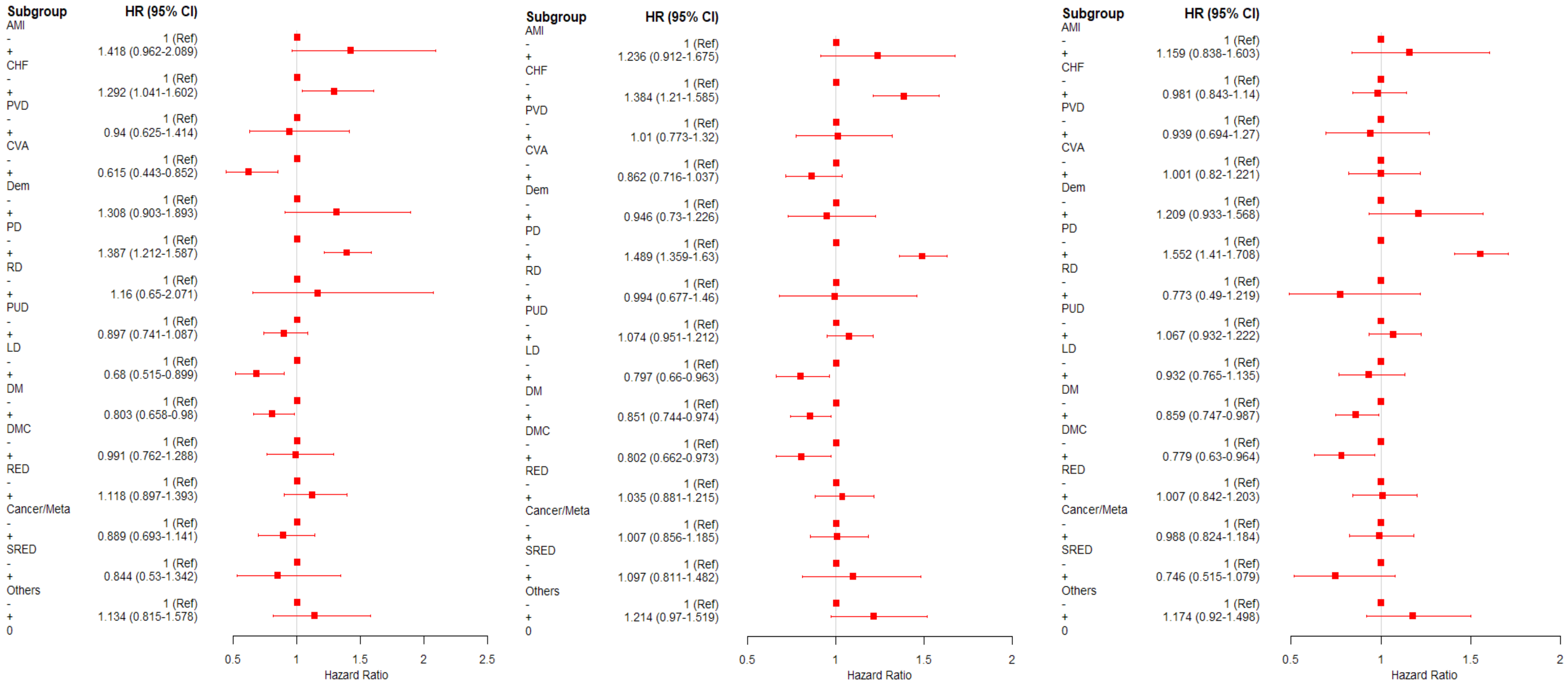

Supplemental Table 1. Sensitivity analysis. Hazard ratios for 5-year all-cause mortality and moderate-to-severe exacerbation

(A) The hazard ratios (HRs) for 5-year all-cause mortality

	Main result (Model 1)	Model 2
GOLD A	Ref	Ref
GOLD B	1.332 (1.244-1.426), p<0.0001	1.338 (1.250-1.433), p<0.0001
GOLD E	1.727 (1.605-1.858), p<0.0001	1.75 (1.626-1.884), p<0.0001

Model 1: Hazard ratios are adjusted for age, gender, BMI, smoking status, COPD family history, CCI, airflow limitation severity, wheezing; Model 2: age, gender, BMI, smoking status, COPD family history, CCI, airflow limitation severity, level of healthcare facilities.

(B) The hazard ratios (HRs) for moderate-to-severe exacerbation

	Main result (Model 1)	Model 2
GOLD A	Ref	Ref
GOLD B	1.407 (1.292-1.532), p<0.0001	1.282 (1.199-1.382), p<0.0001
GOLD E	2.127 (1.942-2.330), p<0.0001	1.921 (1.782-2.071), p<0.0001

Model 1: Hazard ratios are adjusted for age, gender, BMI, smoking status, COPD family history, CCI, airflow limitation severity, wheezing; Model 2: age, gender, BMI, smoking status, COPD family history, CCI, airflow limitation severity, level of healthcare facilities.

Supplemental Table 2. Hazard ratios for 5-year all-cause mortality and moderate-to-severe exacerbations using a time-varying covariate cox proportional hazards model.

(A) The hazard ratios (HRs) for 5-year all-cause mortality

	Main result	Cox model with time-vary covariate
GOLD A	Ref	Ref
GOLD B	1.332 (1.244-1.426), p<0.0001	2.505 (1.956-3.208), p<0.0001
GOLD E	1.727 .605-1.858), p<0.0001	6.205 (3.855-9.988), p<0.0001

(B) The hazard ratios (HRs) for moderate-to-severe exacerbation

	Main result	Cox model with time-vary covariate
GOLD A	Ref	Ref
GOLD B	1.407 (1.292-1.532), p<0.0001	2.517 (2.048-3.095), p<0.0001
GOLD E	2.127 (1.942-2.330), p<0.0001	7.466 (5.088-10.955), p<0.0001

Supplemental Table 3. Hazard ratios for 5-year all-cause mortality and moderate-to-severe exacerbation stratified by healthcare facility level

(A) The hazard ratios (HRs) for 5-year all-cause mortality

	Main result	stratified analysis
GOLD A	Ref	Ref
GOLD B	1.332 (1.244-1.426), p<0.0001	1.303 (1.217-1.394), p<0.0001
GOLD E	1.727.605-1.858), p<0.0001	1.702 (1.582-1.832), p<0.0001

Hazard ratios are adjusted for age, gender, BMI, smoking status, COPD family history, CCI, airflow limitation severity, wheezing

(B) The hazard ratios (HRs) for moderate-to-severe exacerbation

	Main result	stratified analysis
GOLD A	Ref	Ref
GOLD B	1.407 (1.292-1.532), p<0.0001	1.246 (1.161-1.338), p<0.0001
GOLD E	2.127 (1.942-2.330), p<0.0001	1.919 (1.782-2.068), p<0.0001

Hazard ratios are adjusted for age, gender, BMI, smoking status, COPD family history, CCI, airflow limitation severity, wheezing
